# Ceftazidime–Avibactam in Combination with Imipenem as Salvage Therapy for ST11 KPC-33-Producing *Klebsiella pneumoniae*

**DOI:** 10.3390/antibiotics11050604

**Published:** 2022-04-29

**Authors:** Li Ding, Siquan Shen, Renru Han, Dandan Yin, Yan Guo, Fupin Hu

**Affiliations:** 1 Institute of Antibiotics, Huashan Hospital, Fudan University, Shanghai 200040, China; ding_li@fudan.edu.cn (L.D.); 20211220046@fudan.edu.cn (S.S.); hanrenru@fudan.edu.cn (R.H.); yindandan@fudan.edu.cn (D.Y.); guoyan@fudan.edu.cn (Y.G.); 2 Key Laboratory of Clinical Pharmacology of Antibiotics, Ministry of Health, Shanghai 200040, China

**Keywords:** KPC-2, KPC-33, ceftazidime–avibactam, imipenem

## Abstract

A 22-year-old man, after a hematopoietic stem cell transplant, suffered long-term pneumonia caused by *bla*_KPC-2_-positive *K. pneumoniae* and *bla*_KPC-33-_positive *K. pneumoniae* alternately and finally achieved pathogenic clearance and improvement of clinical infectious conditions after using ceftazidime–avibactam in combination with imipenem as salvage therapy. This case provides a reference for treating infection caused by *K. pneumoniae* with a KPC variant in countries lacking new antimicrobial agents.

## 1. Introduction

With the widespread use of ceftazidime–avibactam, KPC-variant-producing *Klebsiella pneumoniae* resistant to ceftazidime–avibactam continues to be identified in clinics worldwide [1,2]. NCBI data show that more KPC variants originating from *bla*_KPC-2_ or *bla*_KPC-3_ were reported in 2020 than in the previous seventeen years combined [3]. A variety of factors tend to mislead clinical anti-infective therapy, including the fact that conventional carbapenemase assays are often falsely negative when detecting KPC variants [4], that strains are often resistant to ceftazidime–avibactam but regain susceptibility to carbapenems such as imipenem, as well as the fact that ESBL phenotypic tests are often positive. There are no uniform recommendations for treating infections caused by KPC-variant-producing strains, especially in countries where new effective antimicrobial agents such as meropenem–vaborbactam are unavailable [4]. Taken together, this requires vigilance against the emergence of KPC variants during therapy which would result in therapy failure. 

Here, we report a case of long-term pneumonia caused by alternating *bla*_KPC-2_-positive *K. pneumoniae* and *bla*_KPC-33_-positive *K. pneumoniae* in a patient with a severe hematological disease who achieved eventual pathogenic clearance and improvement of clinical infectious conditions after using ceftazidime–avibactam in combination with imipenem as salvage therapy.

## 2. Case Presentation

A 22-year-old man was diagnosed with non-Hodgkin lymphoma in December 2020 at a local hospital and underwent a hematopoietic stem cell transplant in July 2021. The patient developed fever, cough, and sputum one week after transplant, while carbapenem-resistant *K. pneumoniae* (CRKP), carrying *bla*_KPC-2_, were isolated from blood, sputum, and rectal swabs, respectively. After being diagnosed with bloodstream infection and pneumonia, he was given anti-infective treatment with meropenem (1 g q8h), polymyxin B (100 mu q12h), tigecycline (100 mg q12h), ceftazidime–avibactam (2.5 g q8h), and other antimicrobial agents successively because of no improvement in clinical symptoms. The patient’s cough and sputum symptoms still did not improve. A lung computed tomography (CT) plain scan showed multiple nodules and patchy shadows in both lungs, which was consistent with the manifestation of pneumonia, so he was admitted to Huashan Hospital affiliated with Fudan University to treat the fever with a cough and sputum in October 2021 (defined as day 1).

After admission, the patient continued anti-infective therapy with ceftazidime–avibactam (2.5 g, q8h, ivgtt) (day 1–day 7). During this time, *Acinetobacter* spp. was isolated from blood culture on day 4. However, since the *Acinetobacter* spp. strain was susceptible to ceftazidime–avibactam (MIC = 4 mg/L), the anti-infective regimen was not changed. However, he still had a cough and sputum, and CT showed a slight improvement in lung inflammation compared to the previous CT scan. On day 6, ceftazidime–avibactam-resistant CRKP (named *K. pneumoniae* HS01) was isolated from a sputum specimen, confirmed using the MALDI-TOF/MS system (bioMérieux, France), which was susceptible to imipenem and tigecycline (Table 1). While the five carbapenemases (KPC, NDM, VIM, IMP, and OXA-48-like) were not detected using the immunochromatographic NG-test Carba 5 assay (NG Biotech, France) in *K. pneumoniae* HS01, the *bla*_KPC-33_ gene was confirmed via polymerase chain reaction and sequencing. On day 8, the anti-infective regimen was switched to imipenem–cilastatin sodium (1 g, q8h, ivgtt) combined with tigecycline (100 mg, q12h, ivgtt) based on the result of antimicrobial susceptibility testing. After one week, the patient’s cough and sputum symptoms improved compared with before.

**Figure 1:**
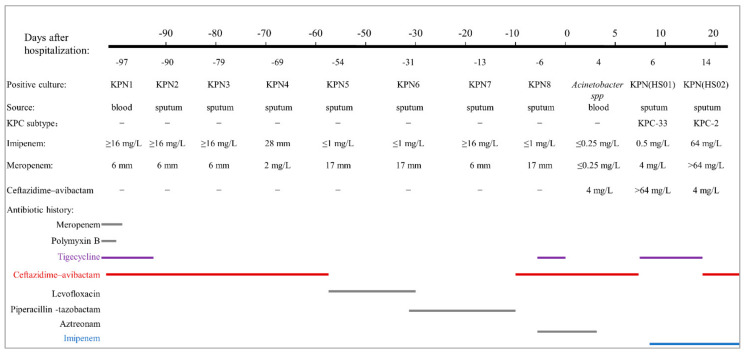
Antibiotic history and the results of microbiology cultures. (The doses of antibiotics are as follows: meropenem, 1 g every 8 h, day 100 to day 94; polymyxin B, 1 million units every 12 h, day 99 to day 97; tigecycline, 100 mg every 12 h, day 99 to day 91, day 4 to day 1, day 7 to day 14; ceftazidime–avibactam, 2.5 g every 8 h, day 97 to day 56, day 9 to day 7, day 15 to day 25; levofloxacin, day 56 to day 32; piperacillin–tazobactam, 4.5 g every 8 h, day 32 to day 9; aztreonam 2 g every 8 h, day 7 to day 1; imipenem 1 g every 8 h, day 8 to day 25).

However, on day 14, the ceftazidime–avibactam-susceptible CRKP (*K. pneumoniae* HS02) was isolated from the sputum specimen, which was susceptible to ceftazidime–avibactam and tigecycline but resistant to imipenem and meropenem (Table 1). KPC was detected using NG-test Carba 5, and PCR and DNA sequencing confirmed *bla*_KPC-2_. According to different treatment plans, the *K. pneumoniae* may switch its corresponding dominant subtype (*bla*_KPC-2_-positive or *bla*_KPC-33_-positive isolate) as needed, resulting in treatment failure. The antimicrobial regimen was changed again to achieve the simultaneous treatment and prevention of dominant clone switching. Ceftazidime–avibactam (2.5 g q8h, ivgtt, to treat the infection caused by *bla*_KPC-2_-positive *K. pneumoniae*) combined with imipenem (1 g q8h, ivgtt, to prevent switching to *bla*_KPC-33_-positive *K. pneumoniae*) was started. The patient then showed significant improvement in cough and sputum symptoms. On days 21 and 23, CT showed an attractive shadow compared with the previous one, and the sputum culture for *K. pneumoniae* was negative. On day 23 after admission, the patient ended this phase of anti-infective treatment and recovered (Figure 1).

Eight *K. pneumoniae* strains (KPN1~KPN8) were isolated from the patient in the local hospital. Though genomic information on these strains was not available, we can infer that KPN 1, KPN 2, KPN 3, and KPN 7 carry *bla*_KPC-2_, and KPN 4, KPN 5, KPN 6, and KPN 8 carry *bla*_KPN-33_ based on results of antimicrobial susceptibility testing. Whole-genome sequencing analysis revealed that *K. pneumoniae* HS01 and *K. pneumoniae* HS02 belonged to ST 11 and carried the same antibiotics resistance genes (Table 1). However, *K. pneumoniae* HS01 carried *bla*_KPC-33_, while *K. pneumoniae* HS02 carried *bla*_KPC-2_. These results suggest that *bla*_KPC-33_-carrying *K. pneumoniae* evolved from *bla*_KPC-2_-carrying *K. pneumoniae*. 

## 3. Discussion

Carbapenem-resistant *Enterobacterales* (CREs) have been ranked as urgent critical threats by WHO due to their high resistance rate and high mortality rate [5]. Clinical studies showed that the mortality rate of CRKP bloodstream infections has reached over 50%, which is 2–3 times higher than that of carbapenem-susceptible *K. pneumoniae* [6]. Ceftazidime–avibactam is favored as a first-line anti-infective agent for treating CRKP infections because it can inhibit the activity of class A, C, and some class D carbapenemases [7]. Unfortunately, the rapid emergence of KPC variants, which undergo amino acid substitutions or insertions at key sites compared to KPC-2 or KPC-3, rendering strains resistant to ceftazidime–avibactam, has posed a new challenge in healthcare facilities [2].

Of note is that patients treated with ceftazidime–avibactam against infection for about two weeks have a significant risk factor for the emergence of the KPC variant [1,2]. The reason for the resistant strains’ emergence after using ceftazidime–avibactam is unclear, although it is speculated that it may be related to the inadequate dose of avibactam. Thus, it is essential to send specimens for microbiological culture and antimicrobial susceptibility testing on time and several times when using ceftazidime–avibactam as an anti-infective therapy to keep track of the dynamic changes in KPC in patients. The treatment of KPC-variant-producing *K. pneumoniae* infection is currently unclear. Data in vitro show the excellent antibacterial activity of meropenem–vaborbactam against KPC-variant-producing *K. pneumoniae* [8]. Sporadic case reports have demonstrated the success of meropenem–vaborbactam in treating *bla*_KPC-31_-positive *K. pneumoniae* infections [9]. Unfortunately, meropenem–vaborbactam is currently only available in a few countries. At the same time, new subtypes of KPC have been detected in several regions and countries, and effective anti-infective treatment options are urgently needed [1,2]. It is noteworthy that tigecycline had excellent activity against *bla*_KPC-33_ carrying *K. pneumoniae* in vitro but had little effect in vivo. This may be related to the dose and duration of treatment in this case. There are few reports on the use of tigecycline in treating KPC-variant-producing *K pneumoniae* infections, and further validation is still needed.

Interestingly, most KPC-variant-producing *K. pneumoniae* regained susceptibility to meropenem or imipenem, suggesting a potential therapeutic role for these antibiotics. Of the nine cases of KPC-variant-producing *K. pneumoniae* infections treated with carbapenems or in combination with other antimicrobial agents, five diseases were successfully cured [2,10,11,12,13,14]. However, in the present case, after the discontinuation of ceftazidime–avibactam and use of imipenem in combination with tigecycline for KPC-33-producing *K. pneumoniae* (imipenem MIC = 0.5 mg/L), KPC-2-carrying *K. pneumoniae* quickly reverted to the dominant clone, failing to use imipenem as an alternative treatment regimen. Not coincidentally, a report by Shi et al. [2] similarly showed the failure of imipenem as salvage therapy for KPC-33-producing *K. pneumoniae* when the KPC-2 clone reappeared in the patient after treatment with imipenem, which may be related to the presence of both KPC-2 as well as KPC-33 clones in the patient. However, KPC variants are often ignored because most carbapenemase phenotypic methods fail to detect KPC variants. Ding et al. [4] reported that KPC variants were not detected using two carbapenemase phenotypic methods (the modified carbapenem inactivation method and the 3-aminophenyl boronic and EDTA method), including KPC-33, KPC-35, KPC71, KPC-76, KPC-78, and KPC-79. Furthermore, clinical laboratories encountering strains that have specific resistance phenotypes (for example, a strain harboring *bla*_KPC-33_ was resistant to ceftazidime–avibactam but susceptible to imipenem) and that are carbapenemase-negative when using carbapenemase phenotypic methods should further define the resistance mechanism by using sequencing to identify possible genetic subtypes. The combination regimen of ceftazidime–avibactam and imipenem was ultimately successful in this case. The combination of ceftazidime–avibactam and imipenem was able to kill both *bla*_KPC-2_-positive *K. pneumoniae* and *bla*_KPC-33_-positive *K. pneumoniae*, blocking the possibility of repeated substitution between *bla*_KPC-2_ and *bla*_KPC-33_. In vitro data similarly showed that the combination of ceftazidime–avibactam and imipenem effectively prevented the emergence of a KPC-variant-resistant subgroup of KPC-3-producing *K. pneumoniae* [15]. However, whether the combination of ceftazidime–avibactam and carbapenems is effective in preventing the emergence of KPC-variant-resistant strains in vivo has not been conclusively established, and the successful combination of ceftazidime–avibactam and imipenem, in this case, may provide a reference for countries where new antimicrobial agents such as meropenem–vaborbactam are not yet available.

The emergence of carbapenemase-producing *K. pneumoniae* has created a “superstorm” in clinical anti-infective therapy worldwide due to its widespread resistance profile. The emergence of KPC variants has undoubtedly added to this, especially in countries with no new effective antibacterial agents. Because routine laboratory testing methods often produce false-negative results when detecting new KPC variants, they may be considered carbapenemase-negative *K. pneumoniae*. During the evolution from *bla*_KPC-2_ to *bla*_KPC-33_ and other new subtypes, if clinicians abandon ceftazidime–avibactam based on the results of antimicrobial susceptibility testing and choose other antimicrobial agents, this may delay anti-infective therapy. Therefore, innovative clinical thinking is needed to treat infections caused by these emerging strains. In this case, although the strain was resistant to ceftazidime–avibactam, ceftazidime–avibactam was still used in order to prevent the next-step mutation according to the evolutionary characteristics of the bacteria. Finally, the combination of ceftazidime–avibactam and imipenem successfully eliminated the bacteria and restored the patient’s health.

## Figures and Tables

**Table 1:** Antimicrobial susceptibility and resistance genes of *K. pneumoniae* HS01 and *K. pneumoniae* HS02.

Antimicrobial Agents	MICs (mg/L) and Resistance Genes			
	*K. pneumoniae* HS 01		*K. pneumoniae* HS 02	
Amikacin	>128	R	>128	R
Ceftazidime	>32	R	>32	R
Cefepime	>128	R	>128	R
Aztreonam	>128	R	>128	R
Meropenem	4	R	>64	R
Imipenem	0.5	S	64	R
Piperacillin–Tazobactam	>256	R	>256	R
Cefoperazone–Sulbactam	>128	R	>128	R
Ceftazidime–Avibactam	>64	R	4	S
Aztreonam–Avibactam	4	S	2	S
Meropenem–Vaborbactam	4	S	16	R
Imipenem–Relebactam	0.25	S	1	S
Cefiderocol	8	I	2	S
Levofloxacin	>16	R	>16	R
Ciprofloxacin	>8	R	>8	R
Tigecycline	2	S	2	S
Polymyxin B	>16	R	>16	R
NG-test Carba 5	Negative		Positive	
β-lactams	*bla*_CTX-M-65_, *bla*_LAP-2_, *bla*_SHV-12_, *bla*_TEM-1B_, *bla*_KPC-33_		*bla*_CTX-M-65_, *bla*_LAP-2_, *bla*_SHV-12_, *bla*_TEM-1B_, *bla*_KPC-2_	
Quinolone	*qnrS1*		*qnrS1*	
Aminoglycoside	*addA2*		*addA2*	
Fosfomycin	*fosA*		*fosA*	
Phenicol	*catA2*		*catA2*	
Trimethoprim	*dfrA14*		*dfrA14*	

R, resistant; S, susceptible.

## Data Availability

The data used during the current study are available from the corresponding author on reasonable request. The bacterial genome sequences have been uploaded to NCBI with the following accession numbers: *K. pneumoniae* HS 01, JAKWJN000000000, *K. pneumoniae* HS 02, JAKWJM000000000.
